# Mitochondrial Transplantation as a Therapeutic Strategy for Inherited Mitochondrial Diseases

**DOI:** 10.1002/advs.202523368

**Published:** 2026-01-22

**Authors:** Parmeshar Singh, Amir Tahavvori, Cyrus E. Kuschner, Blanca B. Espin, Jacob Kazmi, Sofhia V. Ramos, Tai Yin, Kei Hayashida, Kanako Ito‐Hagiwara, Yusuke Endo, Keitaro Yoshioka, Jun Hagiwara, Avijot Sohi, Alisha Oropallo, Ghania Haddad, Timmy Li, Lance B. Becker, Junhwan Kim

**Affiliations:** ^1^ Laboratory for Critical Care Physiology Feinstein Institutes for Medical Research Northwell Health Manhasset NY USA; ^2^ Elmezzi Graduate School of Molecular Medicine Northwell Health Manhasset NY USA; ^3^ Donald and Barbara Zucker School of Medicine at Hofstra/Northwell Health Hempstead NY USA; ^4^ Department of Surgery Comprehensive Wound Healing Center and Hyperbarics Northwell Health Lake Success NY USA; ^5^ Department of Emergency Medicine North Shore University Hospital Manhasset NY USA

**Keywords:** chronic diseases, genetic diseases, mitochondrial transplantation, therapeutics

## Abstract

Mitochondria are essential organelles responsible for cellular energy production and diverse metabolic processes. Mitochondrial dysfunction is implicated in a wide range of diseases. Specifically, genetic mitochondrial diseases, arising from mutations in mitochondrial or nuclear DNA, lead to significant mitochondrial deficits, which result in debilitating and often life‐threatening symptoms. Conventional treatments frequently fail to address these underlying mitochondrial defects, leaving few therapeutic options. Mitochondrial transplantation (MTx), an emerging therapeutic approach involving the delivery of healthy exogenous mitochondria to target cells, has demonstrated beneficial effects in various mitochondria‐mediated diseases in both preclinical and early clinical studies. However, its application to inherited mitochondrial disorders remains largely unexplored and raises important questions about the need for repeated or continuous administration to sustain therapeutic effects. This review systematically examines the potential of MTx for inherited mitochondrial disorders by classifying these diseases by mitochondrial and nuclear DNA origin, critically assessing MTx evidence and mechanisms, and identifying unique translational requirements for chronic inherited disorders. While significant challenges remain, MTx represents a promising approach to directly address mitochondrial dysfunction in these life‐threatening conditions with limited therapeutic alternatives.

## Graphical abstract

Mitochondrial transplantation (MTx) offers a promising therapeutic avenue for mitochondrial diseases. This review comprehensively evaluates MTx, differentiating its feasibility for mtDNA‐ and nDNA‐based disorders. It examines its potential for genetic correction, alongside inherent limitations, technical challenges, and crucial ethical considerations. The review also explores the current landscape and future directions of this innovative strategy.

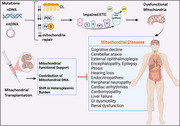

## 1 Introduction

Mitochondria are essential organelles crucial for cellular energy metabolism, generating the bulk of a cell's ATP through the tricarboxylic acid (TCA) cycle, beta‐oxidation, and oxidative phosphorylation [1, 2, 3]. Beyond energy production, mitochondria tightly regulate intracellular calcium (Ca^2+^) levels and reactive oxygen species (ROS) and facilitate essential metabolic processes, making their integrity vital for cellular survival and function [4, 5]. Given their central role in cellular physiology, mitochondrial dysfunction has profound consequences, contributing to the pathogenesis of a wide range of human diseases [6].

From an etiological perspective, mitochondrial dysfunction can be broadly classified into primary and secondary forms and this distinction consequently determines molecular targets and therapeutic strategies [7]. Primary mitochondrial diseases are mainly caused by mutations/deletions in either mitochondrial DNA (mtDNA) or nuclear DNA (nDNA) that encode mitochondrial proteins [7, 8], directly disrupting essential mitochondrial processes [9, 10]. In contrast, secondary mitochondrial dysfunction could arise from extrinsic insults which disrupt mitochondrial homeostasis through mechanisms such as oxidative stress, immune activation, or inflammatory responses [6, 11].

The broad and critical role of mitochondria in cellular health and disease has prompted extensive investigation of mitochondria‐targeting therapies [5, 12]. Although some pharmacological approaches have shown promise in specific contexts, such as certain cancers and neurodegenerative conditions [13, 14], their broader therapeutic application remains constrained by challenges in achieving selective mitochondrial targeting, ensuring safety, and overcoming cellular delivery barriers [15]. These limitations are particularly pronounced in primary mitochondrial diseases where genetic defects inherently compromise mitochondrial structure and function, making conventional pharmacological interventions largely ineffective at addressing the root cause [16, 17]. To date, there are no definitive therapies for mitochondrial dysfunction [6, 8, 17].

Mitochondrial transplantation (MTx) represents an innovative therapeutic strategy that directly addresses these limitations by delivering viable exogenous mitochondria to restore or supplement impaired mitochondrial function [18, 19]. MTx has demonstrated its therapeutic potential by enhancing survival and organ functions across diverse preclinical models [20]. Furthermore, MTx has been explored in early clinical studies, yielding initial insights into its safety, potential efficacy, and early patient responses [21, 22]. Together, these preclinical and early human findings highlight MTx's promising safety and preliminary efficacy, supporting its potential for further clinical development.

However, its utility in diseases caused by primary mitochondrial defects, such as inherited genetic mitochondrial disorders, remains largely unexplored. Unlike acquired mitochondrial dysfunction, these genetic disorders present unique challenges due to the persistent expression of pathogenic mutations and the progressive accumulation of defective organelles across multiple tissue types [7, 8, 9, 10]. The potential requirement for sustained mitochondrial engraftment in the context of ongoing cellular turnover and the need to achieve therapeutic delivery across diverse organ systems necessitate careful consideration of MTx strategies in these genetic conditions.

This review discusses MTx as a potential therapy for genetic mitochondrial diseases. We first provide a detailed view of primary mitochondrial diseases arising from both mtDNA and nDNA mutations, highlighting their molecular basis. We then summarize current preclinical and clinical evidence, detail its potential mechanisms, outline routes and cargo for administration and delivery, and address general safety considerations and challenges associated with MTx. Finally, we explore the potential of MTx as a promising therapeutic avenue for these life‐threatening disorders, where effective strategies to correct mitochondrial dysfunction are limited.

## 2 Genetic Mitochondrial Diseases: An Overview

Primary mitochondrial diseases mainly stem from mutations in both mtDNA and nDNA that encode proteins essential for maintaining mitochondrial homeostasis and function. They are characterized by profound phenotypic heterogeneity, where a single mutation can result in a wide spectrum of symptoms, often affecting tissues that are highly dependent on aerobic metabolism, such as the brain, heart, and muscles. Furthermore, many distinct disorders share overlapping clinical features. Currently, no curative treatments for these diseases exist, and they are often relentlessly progressive with high morbidity and mortality. The overall prevalence of childhood‐onset (<16 years of age) is 5 – 15 cases per 100 000 individuals [23]. The following sections will introduce several key genetic mitochondrial diseases, categorized by whether they arise from mutations in mtDNA or in nDNA or both.

### 2.1 Disorders Primarily Due to Mitochondrial DNA Mutations

**FIGURE 1:**
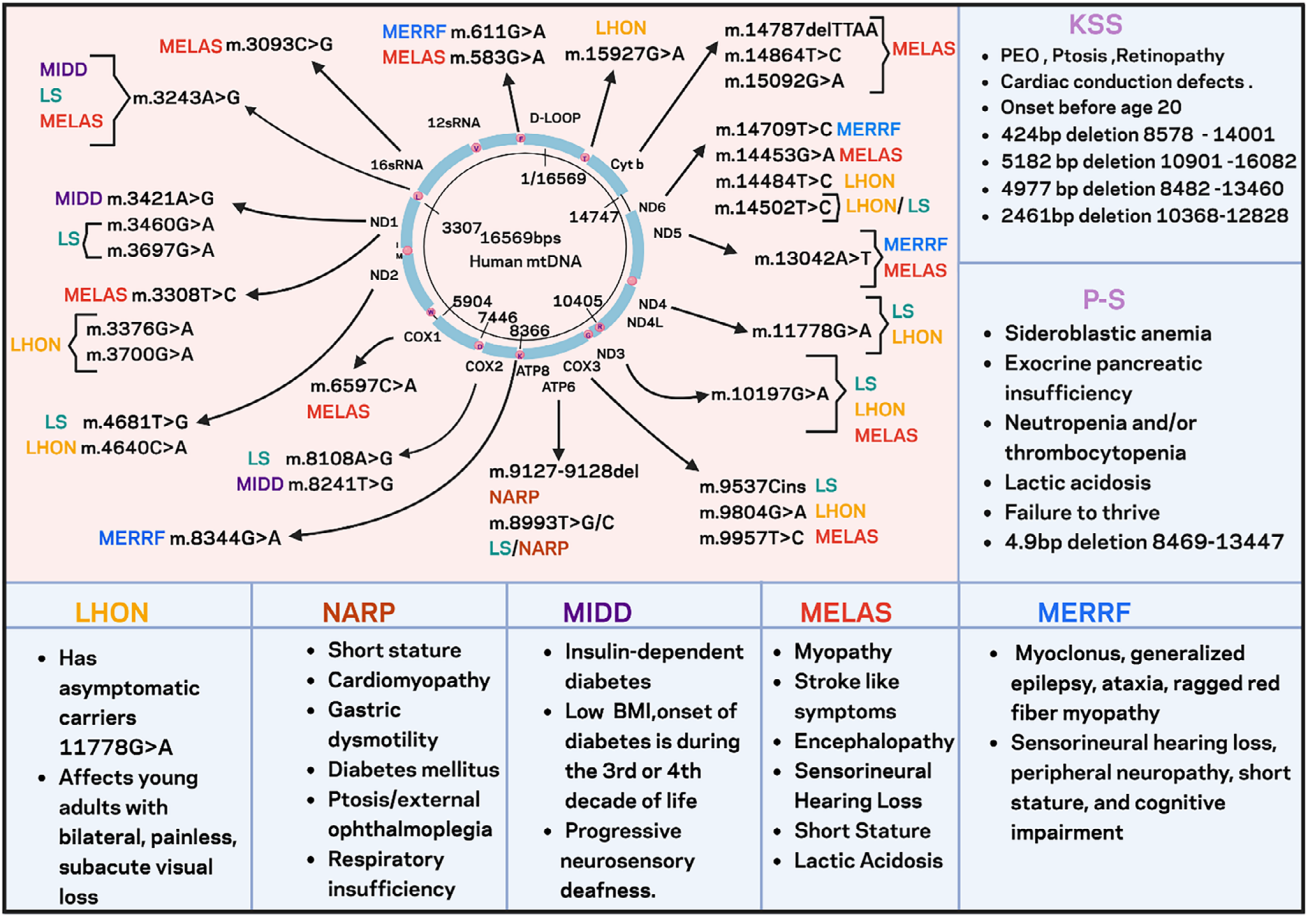
Clinical manifestations and mutations of mitochondrial DNA‐associated disorders. Schematic representation of the human mitochondrial genome (16 569 bp) highlighting common pathogenic mtDNA mutations and brief clinical symptoms. Color‐coding indicates disease phenotype linked to specific/overlapping mutation.

The human mitochondrial genome is a maternally inherited 16 569 base pair circular DNA molecule encoding 37 genes for oxidative phosphorylation: 13 polypeptides of the electron transport chain, 2 ribosomal RNAs, and 22 transfer RNAs [24]. The D‐loop, a non‐coding region, regulates mtDNA replication and gene expression. Unlike nDNA, mtDNA exists in multiple copies per cell and mutations can affect varying proportions of these copies, a phenomenon known as heteroplasmy. The percentage of mutant mtDNA significantly influences disease severity and phenotypic expression. Specific examples of these disorders are detailed below (Figure 1).

#### 2.1.1 Single Large‐Scale Mitochondrial DNA Deletion Syndromes

Single large‐scale mtDNA deletion syndromes (SLMDSs) are characterized by substantial mtDNA loss, presenting as Kearns‐Sayre syndrome (KSS), Pearson syndrome (PS), or progressive external ophthalmoplegia (PEO). These clinically overlapping phenotypes stem from a shared molecular etiology, yet exhibit variable expression, with deletion size and heteroplasmy levels differing significantly even among patients with similar clinical manifestations [25].

With a prevalence of 1–3 per 100 000 [26], KSS typically presents before age 20, featuring progressive external ophthalmoplegia, ptosis, and pigmentary retinopathy. Common accompanying features include cerebellar ataxia, cardiac conduction blocks, hearing loss, short stature, cognitive decline, and endocrinopathies [27]. PS, with a prevalence of approximately 1 in 1 000 000, is often lethal in early childhood [28, 29]. About half of cases stem from a common ∼5‐kilobase deletion, also found in KSS and PEO patients [29, 30]. PS typically presents with hypogenerative anemia, neutropenia, and thrombocytopenia, followed by exocrine pancreatic dysfunction and multi‐organ complications. Anemia may spontaneously resolve due to improved heteroplasmy, though other symptoms persist [31, 32]. Metabolic dysfunction, including lactic acidosis is also common [33]. PEO mainly results from large mtDNA deletions of about several kilobases that typically affect the major arc region (around nucleotides 7 800–15 500) [34]. Clinically, PEO is characterized by gradually progressive and bilateral ptosis and impaired ocular mobility while preserving pupil function, without remission or exacerbations [35].

Treatment strategies for these mitochondrial diseases include gene therapy, pharmacological enhancement of mitochondrial function (such as clinical trials of EPI‐743, a Coenzyme Q10 (CoQ10) analog), and other novel approaches [36, 37]. For instance, hematopoietic stem cell transplantation for anemia [38], adeno‐associated virus‐mediated gene transfer for rare nuclear gene‐linked PEO models (though unsuitable for most SLMDSs) [39], and pronuclear transfer, a method that reduces SLMDS transmission by transferring nDNA to healthy donor eggs, resulting progeny exhibiting reduced heteroplasmy [40]. A pioneering approach is mitochondrial augmentation therapy. This method involves using autologous CD34+ cells, ex vivo enriched with maternal mitochondria before bone marrow transplantation, which has demonstrated improved patient outcomes including reduced heteroplasmy and enhanced strength [41, 42, 43], likely mediated by natural mitochondrial transfer mechanisms.

#### 2.1.2 Leber's Hereditary Optic Neuropathy

Leber's hereditary optic neuropathy (LHON) is a progressive ophthalmological disorder caused by point mutations in complex I subunit genes. Three mutations account for almost 90% of known cases: m.11778G>A (*MT‐ND4*), m.3460G>A (*MT‐ND1*), and m.14484T>C (*MT‐ND6*) [44, 45]. The frequency of LHON ranges between 1 in 31 000–50 000 and is typically more common in males. Onset begins in late adolescence to early adulthood with central vision loss, starting in one eye and progressing to the second within weeks, owing to the loss of retinal ganglion cells that form the papillomacular bundle [46]. Associated symptoms include cardiac conduction blocks or minor focal neurological deficits, with the m.14484T>C (*MT‐ND6*) mutation linked to the highest chances of spontaneous recovery [46]. mRNA‐loaded nanoparticle‐engineered mitochondria (mNP‐Mito) have shown success in restoring mitochondrial function in cell culture and animal models of LHON [47, 48].

#### 2.1.3 Mitochondrial Encephalopathy, Lactic Acidosis, and Stroke‐Like Episodes

Mitochondrial Encephalopathy, Lactic Acidosis, and Stroke‐Like Episodes (MELAS) is a progressive neurodegenerative disorder. About 80% of cases are linked to the m.3243A>G mutation in the *MTTL1* gene that encodes tRNA^Leu/(UUR)^ with a mean onset age of 8.1 years [49, 50, 51]. The prevalence ranges from 0.6 to 16 per 100 000 individuals [52, 53, 54]. Characteristic manifestations include encephalopathy, lactic acidosis, and stroke‐like episodes, reflecting the syndrome's broad impact on high‐energy‐demand tissues [55, 56].

Management centers on improving mitochondrial bioenergetics and minimizing oxidative stress using agents such as riboflavin, CoQ10, creatine monohydrate, L‐carnitine, bezafibrate, KH176, and high‐dose taurine supplementation [57]. Nitric oxide (NO) enhancers (e.g., arginine, citrulline) have also been tested [58, 59]. Further research explores immunonutrition, stem cell transplantation, and gene therapies [59, 60, 61, 62, 63].

#### 2.1.4 Myoclonic Epilepsy with Ragged‐Red Fibers

Myoclonic Epilepsy with Ragged‐Red Fibers (MERRF) is primarily caused by mtDNA mutations that disrupt aminoacylation, impairing mitochondrial protein synthesis. This leads to the characteristic clinical manifestations of the syndrome, such as myoclonic epilepsy, ataxia, and myopathy [64, 65, 66, 67]. The m.8344A>G mutation in the mitochondrial tRNA^Lys (*MTTK*) gene accounts for approximately 80%–90% of cases [68]. Histologically, ragged‐red fibers (subsarcolemmal mitochondrial accumulations) are often observed, with myoclonus frequently being the initial symptom [67]. MERRF can also overlap with MELAS, KSS, and Neuropathy, Ataxia, and Retinitis Pigmentosa (NARP) [69, 70, 71], and may include other symptoms such as sensorineural hearing loss, peripheral neuropathy, short stature, and cognitive impairment [72]. Therapeutic strategies focus on seizure control with anticonvulsants and supplements such as CoQ_10_ and vitamins [73, 74]. Rapamycin treatment has shown potential in improving mitochondrial respiration in cells with intermediate heteroplasmy [75]. Patient‐derived iPSC neurons, exhibiting impaired synaptic function and bioenergetic deficits, offer a promising model for novel therapeutic screening [76].

#### 2.1.5 Neuropathy, Ataxia, and Retinitis Pigmentosa

NARP is caused by heteroplasmic mutations in the MT‐ATP6 gene, most notably m.8993T>G/C, typically present at 70%–90% heteroplasmy [77]. NARP is characterized by axonal neuropathy, retinitis pigmentosa, muscle weakness, and cerebellar ataxia, frequently accompanied by seizures, cognitive decline, hearing loss, and cardiac arrhythmias [78]. The clinical spectrum ranges from NARP‐like syndromes to classic NARP, often overlapping with Leigh syndrome, and observed in both early‐onset and late‐onset forms [79]. Diagnosis is established through a combination of clinical assessment and specialized tests, including neuro‐ophthalmologic evaluations such as fundus photography and optical coherence tomography, electrophysiological studies, muscle biopsy, and confirmatory genetic analysis [80]. Management aims to control neurological symptoms and preserve vision.

#### 2.1.6 Maternally Inherited Diabetes and Deafness

Maternally Inherited Diabetes and Deafness (MIDD) is commonly caused by a point mutation m.3243A>G in mtDNA, affecting the mitochondrial tRNA^Leu (UUR) and disrupting oxidative phosphorylation [81, 82]. Between 0.5% to 2.8% of patients with diabetes have MIDD [83, 84]. Sensorineural hearing loss is one of the hallmark features of MIDD, often manifesting in early adulthood. MIDD also involves myopathy, macular dystrophy, cardiomyopathy, renal dysfunction and less commonly neuropsychiatric or gastrointestinal issues [85, 86]. The disease phenotype varies with heteroplasmy levels, ranging from MIDD to MELAS, with about 6% of cases showing MELAS/MIDD overlap [87, 88]. Higher heteroplasmy in liver, kidney, spleen, and leukocytes is associated with a shorter lifespan [89]. Current therapeutic advancements include the long‐term use of dipeptidyl peptidase‐4 inhibitors and sodium‐glucose cotransporter‐2 inhibitors and other approaches include administration of glucagon‐like peptide 1 receptor agonists and mitochondrial supplements like CoQ10 [90].

### 2.2 Disorders Primarily Due to Nuclear Gene Mutations

**FIGURE 2:**
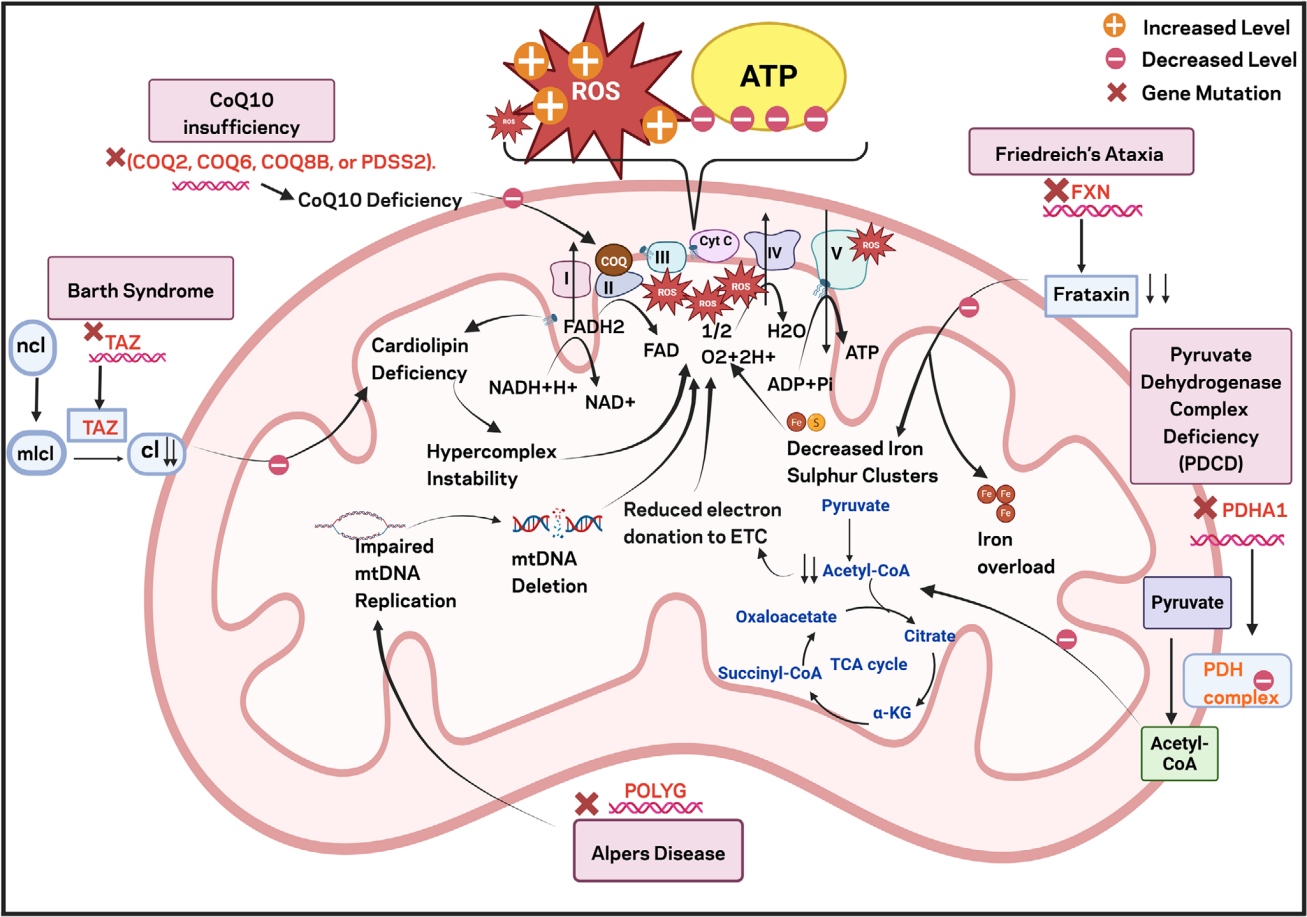
Mitochondrial dysfunction and nDNA associated genetic disorders. This diagram illustrates key mitochondrial disorders and their related genetic causes. Defects in these genes impair electron transport chain activity, oxidative phosphorylation, and mitochondrial lipid remodeling, leading to decreased ATP synthesis and elevated reactive oxygen species (ROS) levels. FXN, Frataxin; PDC/PDH complex, Pyruvate Dehydrogenase Complex; POLG, DNA Polymerase Gamma; TAZ, Tafazzin; CoQ10, Coenzyme Q10; NCL, Nascent Cardiolipin; CL, Cardiolipin; MLCL, Monolysocardiolipin.

While mtDNA encodes only 13 essential polypeptides of the electron transport chain, the vast majority of mitochondrial proteins, over 1,500 in mammals, are encoded by nDNA [91]. Consequently, mutations in these nuclear genes can lead to a wide spectrum of mitochondrial disorders, often presenting with clinical features overlapping those of mtDNA‐based diseases (Figure 2). This section details key examples of disorders primarily caused by nDNA mutations.

#### 2.2.1 Barth Syndrome

Barth syndrome (BTHS) is an X‐linked mitochondrial disorder caused by mutations in the tafazzin (*TAZ)* gene. TAZ protein is essential for biosynthesis of cardiolipin, a crucial phospholipid for mitochondrial structure and function [92, 93, 94]. Therefore, *TAZ* mutations lead to defective cardiolipin remodeling, which compromises oxidative phosphorylation and ultimately reduces ATP generation [95]. With the prevalence of roughly 1 in 300 000, BTHS typically manifests with severe symptoms in infancy or childhood, including dilated cardiomyopathy, neutropenia, skeletal myopathy, and neuropsychological and neurophysiological phenotypes [96]. Cardiomyopathy is the leading cause of diagnosis and mortality in BTHS [97, 98]. Within 15 years, one‐fourth of patients with BTHS cardiomyopathy progress to cardiac death or transplant [99]. Patients often experience delayed diagnosis and face unique nutritional and developmental challenges, including growth failure [100, 101, 102, 103, 104, 105]. Treatment for BTHS focuses on managing cardiac dysfunction and arrhythmias, alongside nutritional support to address associated growth delays and metabolic complications [106, 107, 108]. Experimental therapies continue to evolve, including elamipretide (SS31), which recently received FDA accelerated approval, and bezafibrate, a PPAR agonist [109, 110, 111].

#### 2.2.2 Pyruvate Dehydrogenase Complex Deficiency

Pyruvate Dehydrogenase Complex Deficiency (PDCD) results from mutations in genes encoding the enzymes of the pyruvate dehydrogenase complex (PDC). The majority of PDCD cases are caused by mutations in the X‐linked PDHA1 gene, which encodes the pyruvate dehydrogenase E1α subunit (accounting for around 80% of mutations). Other cases arise from mutations in genes for other PDC subunits, E3‐binding protein, or in the gene for PDH phosphatase (PDP1) [112].

These mutations diminish the activity of PDC, which converts pyruvate into acetyl‐CoA; this disruption leads to chronic lactic acidosis and severe developmental delay [113]. Treatment mainly involves dietary changes like a high‐fat, low‐carbohydrate ketogenic diet. Current research also explores agents such as dichloroacetate and phenylbutyrate, with approaches including gene therapy and stem cell research [114, 115].

#### 2.2.3 Friedreich's Ataxia

Friedreich's ataxia (FRDA) is the most common inherited ataxia, caused by GAA repeat expansions in the frataxin gene, resulting in frataxin deficiency, mitochondrial iron accumulation, and excessive ROS generation [116, 117, 118]. Affecting about 3 in 100,000 individuals, FRDA typically presents in adolescence with progressive ataxia, cardiomyopathy, and diabetes, and leads to a shortened lifespan [119, 120]. Cardiac complications are a major cause of mortality [121]. Current therapeutics include the newly approved Omaveloxolone (Skyclarys) [122], agents targeting oxidative stress (Vatiquinone, Elamipretide, Resveratrol), and gene therapy [123, 124, 125, 126, 127].

#### 2.2.4 Alpers–Huttenlocher Syndrome

Alpers–Huttenlocher syndrome (AHS) is caused by mutations in the *POLG* gene, which impairs the mtDNA polymerase responsible for mtDNA replication [128]. It is one of the most devastating POLG‐related disorders, primarily characterized by an early‐onset progressive encephalopathy often accompanied by liver failure, although late onset is also possible [129, 130, 131]. With a prevalence of approximately 1 in 100 000, the disease is usually fatal within the first decade of life [132]. Current management focuses on addressing seizures and other complications. Investigational approaches, such as stem cell and gene therapies, remain in preliminary stages [133, 134, 135].

#### 2.2.5 Coenzyme Q10 Deficiency

CoQ10 is an essential lipid component of the mitochondrial respiratory chain. Primary CoQ10 deficiency includes a group of disorders caused by biallelic pathogenic variants in the genes including COQ2, COQ8A (ADCK3), COQ5, COQ8B (ADCK4), COQ9, PDSS1, PDSS2, COQ6, COQ4, and COQ7, which are involved in the CoQ10 biosynthetic pathway. Novel variants like L122P (c.365T>C, p.Leu122Pro) have also been identified [136]. Clinical phenotypes include mitochondrial encephalomyopathy, cerebellar ataxia, severe infantile multisystemic disease, steroid‐resistant nephrotic syndrome, and isolated myopathy [137]. Symptoms can manifest from the neonatal period to adulthood and even old age [138, 139]. Brain MRI patterns in CoQ10 encephalopathy can overlap with those of MELAS and Leigh syndrome [140]. In contrast, secondary CoQ10 deficiencies arise from gene variants unrelated to CoQ10 biosynthesis or from non‐genetic factors [141, 142]. CoQ10 supplementation remains the primary therapeutic approach [143].

#### 2.2.6 Mitochondrial DNA Depletion Syndromes

MtDNA Depletion Syndromes (MDDS) are autosomal recessive disorders characterized by a significant reduction in mtDNA copy number. This depletion leads to impaired oxidative phosphorylation and organ‐specific dysfunction, most commonly affecting muscle, liver, and brain. MDDS are caused by mutations in nuclear genes involved in mtDNA synthesis and maintenance, such as *TK2, POLG, DGUOK, and MPV17*, with molecular diagnostic advances expanding the list of causative genes to include novel variants like GUK1 [144, 145]. These diagnostic tools are enabling earlier and more accurate diagnosis, particularly in neonates with liver dysfunction [146, 147]. A key therapeutic breakthrough for MDDS has been the use of deoxynucleoside therapy (deoxycytidine and deoxythymidine), which restores mtDNA levels and improves survival in *TK2*‐related MDDS [148, 149]. Concurrently, genotype‐driven interventions and disease‐modifying strategies are also rapidly advancing [150].

### 2.3 Disorders Due to Mitochondrial DNA or Nuclear DNA Mutations

#### 2.3.1 Leigh Syndrome

Leigh syndrome is a progressive neurodegenerative disease characterized by bilateral lesions in the basal ganglia, thalamus, and brainstem [151, 152, 153]. Affecting approximately 1 in 40 000 births, it typically becomes apparent in infancy, though late onset forms also exist [153, 154, 155]. Leigh syndrome carries a poor prognosis, with around 10% of patients surviving by the age of 6 [156, 157]. Current treatment includes supplements like CoQ10, thiamine, idebenone, and pyruvate, alongside gene replacement therapy [158, 159, 160]. Recently, MTx has shown promise in preclinical models of Leigh syndrome, which is discussed in detail later.

## 3 Mitochondrial Transplantation: Current Landscape

MTx combats mitochondrial dysfunction by directly introducing healthy mitochondria into affected cells to restore mitochondrial function. However, it is important to note that MTx is not simply about replacing damaged mitochondria with healthy ones. MTx leverages the unique ability of mitochondria to integrate and operate within host cellular environments, thereby restoring critical mitochondrial functions such as energy production and oxidative balance and further triggering regulatory and signaling effects beyond mere organelle supplementation. The following sections address the growing field of MTx, examining the existing body of preclinical work, MTx delivery strategies, exploring mechanisms of mitochondrial internalization, evaluating clinical status, and assessing safety considerations, all crucial aspects for understanding the full potential and future direction of this innovative treatment.

### 3.1 Therapeutic Applications of Mitochondrial Transplantation in Preclinical Studies: From In Vitro Systems to In Vivo Models

Mitochondria can naturally transfer between cells under both physiological and pathological conditions [161]. This process, known as intercellular mitochondrial transfer, involves the direct donation of mitochondria from one cell to another without cell division [162, 163]. Both normal and malignant cells can release functional mitochondria into the extracellular environment [164, 165, 166]. Researchers have also demonstrated mitochondrial transfer from antibiotic‐resistant cells to sensitive cells, resulting in phenotypic changes in the recipient cells [167].

Leveraging this natural phenomenon, researchers have developed artificial mitochondrial transfer techniques for therapeutic applications. For instance, mitochondria derived from platelets can be internalized by human umbilical vein endothelial cells via endocytosis, providing protection by reducing oxidative stress and limiting apoptotic cell death [168]. Chondrocytes from osteoarthritis patients have been shown to successfully internalize mitochondria derived from umbilical cord–derived mesenchymal stem cells, which promoted mitochondrial fusion, restored mitochondrial dynamics, and protected against oxidative stress and apoptosis [169]. Studies have shown that xenogenic transplantation of mitochondria derived from mouse liver tissue can restore respiratory function in human *ρ* ^0^ cells lacking functional mitochondria [170]. In addition, evidence also suggests that mitochondria from human hepatoma cells can successfully internalize into human neuroblastoma SH‐SY5Y cells, further supporting the feasibility of cross‐cellular mitochondrial transfer [171]. These in vitro studies demonstrate this inter‐cellular mitochondrial transfer, which enhances host cell mitochondrial function, normalizes cellular metabolism, and promotes cell survival under stress conditions.

The therapeutic efficacy of MTx has been extensively validated across diverse preclinical animal models, from small rodents to large mammals, demonstrating therapeutic potential across multiple organ systems and disease pathologies. In the central nervous system, MTx has demonstrated significant neuroprotective effects. In a mouse model of cerebellar neurodegeneration, liver‐derived MTx improved neuronal mitochondrial structure and function, reduced pathology (mitophagy and apoptosis), and alleviated cerebellar ataxia [172]. For Parkinson's disease, MTx reduced microglial inflammation and improved behavioral outcomes in a mouse model [173], while in rat models, both syngeneic and xenogeneic MTx restored mitochondrial function, mitigated oxidative stress, protected dopaminergic neurons, and enhanced motor function [174]. Beyond neurodegenerative conditions, local MTx in a rat spinal cord injury model led to functional recovery by decreasing inflammation, suppressing mitochondrial fragmentation and apoptosis, and protecting against demyelination [175].

In cardiovascular disease models, MTx has shown particular promise in addressing ischemia‐reperfusion injury. In both mouse and rat models of asphyxial cardiac arrest, MTx significantly improved survival and neurological outcomes [176, 177, 178]. In mice, MTx modulated immune and transcriptional responses, contributing to systemic organ protection [177], while in rats it enhanced cerebral perfusion, accelerated restoration of arterial lactate and glucose homeostasis, and reduced lung injury [178]. In rabbit models, administration of mitochondria into regional ischemic area after myocardial ischemia‐reperfusion significantly decreased infarct size and improved postischemic cardiac function as well as mitochondrial function [179]. In large animal models, direct myocardial injection of autologous mitochondria in swine reduced infarct size and improved left ventricular function following ischemia [180]. Similarly, in a swine cardiac ischemia model, autologous MTx significantly enhanced myocardial cell viability following ischemia and reperfusion injury [181]. Notably, in aged rat hearts, the combination of CoQ10 with MTx significantly improved cardiac recovery following ischemia‐reperfusion injury, outperforming either treatment alone by enhancing mitochondrial function, reducing oxidative stress, and decreasing infarct size [182].

MTx has also demonstrated therapeutic efficacy in other organ systems and disease contexts. In a swine acute kidney injury model, intra‐arterial MTx enhanced glomerular filtration, reduced tubular necrosis, and improved overall renal function [183]. In a polymicrobial sepsis rat model, intravenous MTx significantly improved survival, enhanced bacterial clearance, and reduced splenic apoptosis [184]. In rat models of muscle injury, intramuscular MTx restored muscle mass, reduced lactate accumulation, and enhanced myofiber hypertrophy [185]. These broad therapeutic implications of MTx across diverse organ systems and disease contexts offer compelling preclinical evidence to support its development as a clinical therapeutic strategy.

### 3.2 Clinical Application of Mitochondrial Transplantation

**FIGURE 3:**
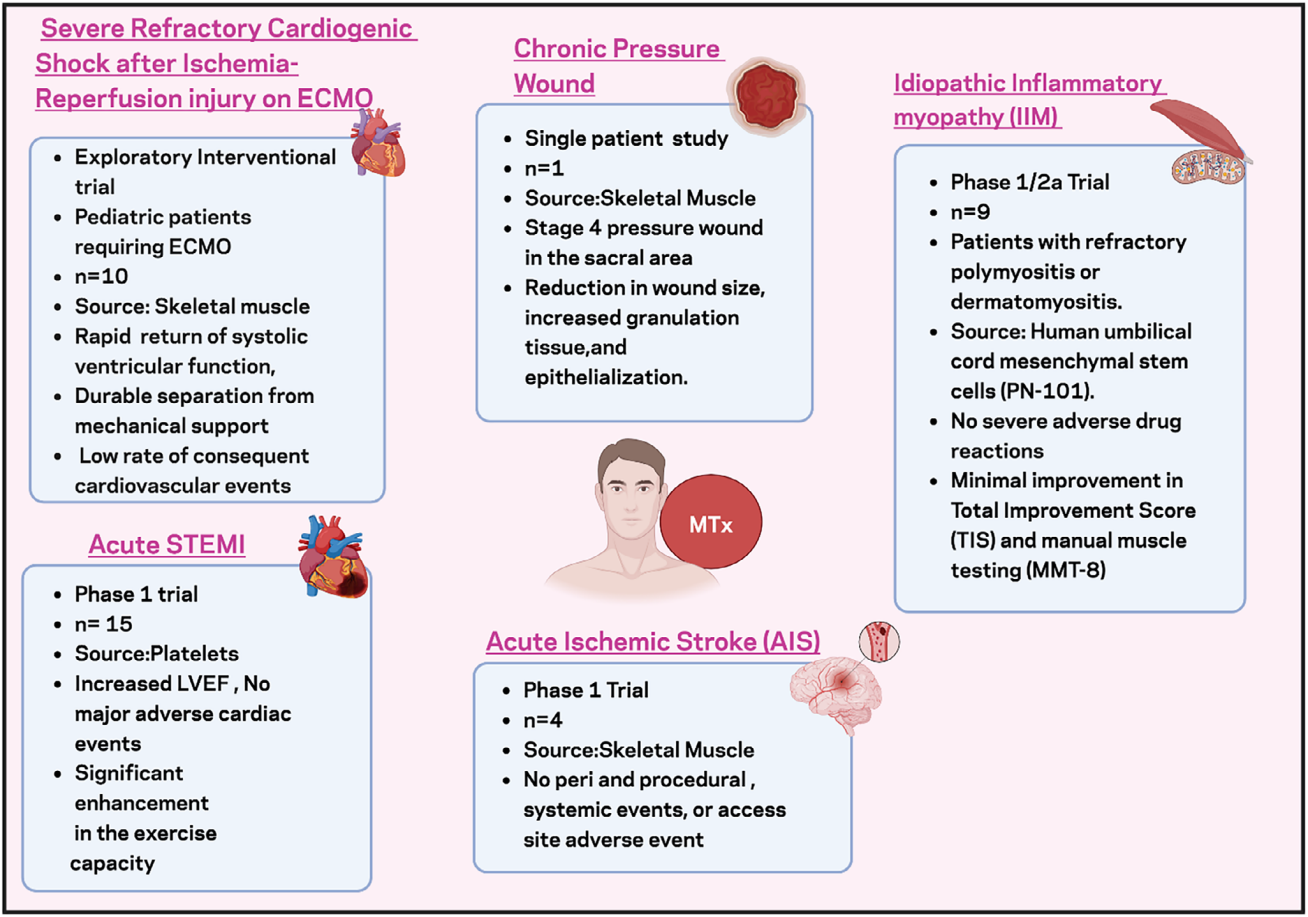
Summary of early clinical mitochondrial transplantation studies. This figure presents an overview of reported clinical studies involving mitochondrial transplantation (MTx) across various medical conditions. Each study includes details on trial phase, sample size (ranging from N = 1 to N = 11), cell source, safety outcomes, adverse events, and therapeutic efficacy. ECMO, extracorporeal membrane oxygenation; LVEF, left ventricular ejection fraction; STEMI, ST‐elevation myocardial infarction.

Clinical studies investigating MTx also have demonstrated promising results across several medical conditions (Figure 3), establishing preliminary evidence for safety and therapeutic potential in human applications. A pioneering clinical application was reported at Boston Children's Hospital, focusing on pediatric patients with cardiac defects. Autologous mitochondria isolated from healthy, non‐ischemic skeletal muscle were injected into pediatric patients on central extracorporeal membrane oxygenation (ECMO) support with injured myocardium following ischemic damage. Four of the five subjects demonstrated improved ventricular function and were successfully weaned from ECMO support, experiencing no short‐term adverse effects [21]. In a prospective, triple‐blinded, parallel‐group, block‐randomized controlled trial in ST‐elevation myocardial infarction, patients receiving platelet‐derived MTx demonstrated notable improvements in exercise capacity and symptom relief compared to controls, without significant adverse cardiac events [22].

Building on these cardiac applications, researchers expanded MTx into neurological conditions. The first study involving direct MTx application in the brain was a Phase 1 trial for acute ischemic stroke that evaluated the safety of integrating autologous MTx into standard stroke treatment. No periprocedural events, post‐procedure events, systemic events, or access site complications were reported. This study presents the first evidence demonstrating the safety potential of MTx to reduce ischemic damage in the human brain [186].

A recent phase 1/2a open‐label, dose‐escalation clinical trial evaluated MTx in 9 patients with refractory polymyositis or dermatomyositis, specific types of idiopathic inflammatory myopathies [187]. These autoimmune conditions are characterized by symmetric proximal muscle weakness and are increasingly recognized to involve mitochondrial dysfunction, evidenced by downregulated mitochondrial genes, anti‐mitochondrial antibodies, and mtDNA deletions [188]. The trial demonstrated modest improvements in total improvement score along with transient reductions in serum creatine kinase levels and stabilization of manual muscle testing scores in several participants. Supporting mechanistic evidence showed that primary myoblasts from IIM patients exhibited defective myogenesis and mitochondrial dysfunction, which were restored following treatment with umbilical cord‐derived MTx. In complementary murine myositis models, MTx significantly suppressed inflammation and restored malate/aspartate ratio [187].

Another experimental application of MTx involved a 22‐year old patient presenting with a pressure ulcer in the sacral region. Skeletal muscle derived MTx resulted in a notable reduction in wound size, improved tissue granulation, and mitigation of progressive necrosis. No debridement was required, and there were no signs of local or systemic infection. Although limited to a single patient, this represents a successful attempt at employing MTx for wound healing in a clinical setting [189]. These studies emphasize the therapeutic ability of MTx in humans, showing improved patient outcomes across diverse diseases with minimal to no major adverse events.

### 3.3 Enhancing Mitochondrial Transplantation Delivery and Tissue Incorporation

As MTx research has expanded from direct organ injection to systemic administration methods and diversified across various diseases, a growing body of research is dedicated to refining mitochondrial delivery approaches with the dual aim of improving cellular uptake and enhancing therapeutic efficacy. Peptide‐based delivery systems have emerged as promising approaches for enhancing mitochondrial uptake. Systems such as peptide‐1 (Pep‐1) and Szeto‐Schiller peptides promote direct mitochondrial internalization, improve retention, and enable targeting of damaged tissues [190, 191]. Magnetically guided mitochondrial transfer also has emerged as a novel precision technique in treating intracerebral hemorrhage [192].

Innovative mechanical and physical delivery methods have also been developed. MitoPunch represents a mechanically driven system that facilitates delivery of isolated mitochondria into mammalian cells lacking mtDNA (ρ^0^ cells), enabling stable incorporation of donor mitochondria and effectively restoring mitochondrial function while providing controlled engineering of cells with defined mitochondrial and nuclear genome pairings [193]. Similarly, photothermal nanoblade technology allows introduction of isolated mitochondria from genetically distinct donors into recipient cells, leading to recovery of metabolic gene expression, cellular bioenergetics, and metabolite balance, while enabling direct assessment of mitochondrial function across different donor types [194]. Moreover, nasal administration of mitochondria has demonstrated functional recovery in models of Parkinson's disease, stroke, and chemotherapy‐induced cognitive decline [6, 195, 196]. Additionally, oral delivery of mitochondria using nanomotors or erythrocyte carriers has shown promising therapeutic effects by enhancing mitochondrial uptake and function in ischemic heart disease and pulmonary hypertension models [197, 198]. These diverse delivery strategies will be highly beneficial as the field advances toward clinical translation, making standardizing these approaches and systematically evaluating their safety profiles critical next steps.

### 3.4 Mitochondrial Transfer and Mechanisms

Despite the well‐documented therapeutic benefits observed with MTx, the precise mechanisms by which transplanted mitochondria exert their effects remain incompletely understood. This complexity arises from the intricate relationship between nuclear and mitochondrial genomes, where mitochondrial function profoundly influences mitochondria‐nuclear crosstalk and nuclear gene expression. A central question is whether the primary benefits stem from direct metabolic support, genetic complementation, or broader cellular protective effects. This critical distinction remains an active area of MTx investigation. While other factors may also contribute, current evidence suggests three prominent possible mechanisms underlying MTx therapeutic efficacy.

#### 3.4.1 Theory 1: Mitochondrial Functional Support

The primary mechanism underlying MTx therapy is the direct support and restoration of impaired mitochondrial function within recipient cells [199]. Mitochondrial disorders caused by mutations in nDNA or mtDNA result in compromised cellular bioenergetics, including decreased ATP production, increased oxidative stress, and activation of cell death pathways [8]. By transferring functional mitochondria, MTx replenishes the recipient cells' mitochondrial pool and integrates into the endogenous mitochondrial network to restore bioenergetics and other essential mitochondrial functions in damaged or metabolically dysfunctional cells [200]. This metabolic rescue improves cellular energy homeostasis and viability, ultimately mitigating tissue injury and promoting recovery. Preclinical studies demonstrate that MTx improves outcomes in diverse acute and chronic conditions, such as ischemic injuries (cardiac, renal, spinal cord), cardiomyopathies, and neurodegenerative diseases like Parkinson's and Alzheimer's. The therapeutic effects are driven by improved oxidative phosphorylation and mitochondrial quality, including enhanced clearance of damaged mitochondria [201, 202]. Thus, supporting impaired mitochondrial function through bioenergetic restoration is the key and foundational mechanism by which MTx exerts many of its beneficial effects [199].

#### 3.4.2 Theory 2: Contribution of Mitochondrial DNA

Natural aging and pathological conditions such as sepsis, ischemia‐reperfusion injury, and chronic inflammation can accelerate mtDNA damage and mutation accumulation, which cumulatively impair mitochondrial function [203, 204]. This deterioration may result from a combination of replication errors, decreased efficiency of DNA repair mechanisms, and diminished mitochondrial quality control processes, including mitophagy. MTx introduces intact exogenous mtDNA, which can compensate for deficits in mitochondrial‐encoded proteins that are absent or dysfunctional due to endogenous mtDNA mutations. Importantly, preclinical in vitro studies have demonstrated that transplanted mitochondria and their mtDNA can be retained within recipient cells for extended periods. For example, transplanted mitochondria were shown to retain their mtDNA features for 12 passages of cells in one study, and for 5 weeks in another when endogenous mtDNA was depleted [167, 205]. More broadly, target cells appear to consistently retain exogenous mtDNA for a period of time following MTx [206, 207, 208, 209]. This retention carries clear mechanistic importance for disorders caused by direct mtDNA alteration, as it facilitates the production of mitochondrial proteins that were previously absent or misformed, thereby returning physiological function and halting further injury. In the case of nDNA pathologies, the retention of exogenous mtDNA may further serve as a bridge, providing fresh stores of intact exogenous mtDNA and reducing the impact of chronic mtDNA injury.

#### 3.4.3 Theory 3: Shift in Heteroplasmic Burden

Mitochondrial genomic disorders pose a unique progression due to the development of heteroplasmy [210]. As maternally inherited mtDNA exists in hundreds to thousands of copies per cell, the proportion of pathogenic to wild‐type mtDNA copies can vary within and between cells. Through processes like mitochondrial fission and fusion, and subsequent cell division, this relative percentage, or heteroplasmic burden, can dynamically shift. Over time, these changes can lead to an accumulation of pathogenic mtDNA copies, eventually surpassing a critical bioenergetic threshold. Once this threshold is crossed, it compromises tissue‐specific mitochondrial function, ultimately leading to end‐organ injury and the chronic, progressive disease characteristic of mtDNA deficits, particularly in high‐energy‐demanding tissues.

One compelling theory for how MTx may treat both nDNA and mtDNA diseases is by directly influencing this heteroplasmic burden. The administration of healthy, exogenous mitochondria and their intact mtDNA could potentially incorporate into the recipient cell's mitochondrial network. This incorporation might then either directly dilute the proportion of mutated mtDNA, or, by introducing new functional mitochondria, enable the cell to avoid or resolve the bioenergetic deficit caused by existing mutations, thereby pushing it back below the disease threshold. Alternatively, MTx might act more broadly by reducing systemic cellular or mitochondrial injury through beneficial metabolic and antioxidant activity. Whether MTx directly aids in shifting the underlying heteroplasmic burden by replacing faulty mtDNA, or primarily provides transient metabolic support that alleviates mitochondrial stress and indirectly delays disease progression, remains a critical area for further investigation.

## 4 Application of MTx to Genetic Mitochondrial Diseases

Unlike conventional pharmacological approaches that struggle with mitochondrial targeting and delivery, MTx bypasses the challenge of crossing mitochondrial membranes by delivering mitochondria to target cells, making it significantly more efficient. Furthermore, MTx addresses multiple pathological mechanisms simultaneously rather than targeting individual symptoms, making it particularly well‐suited for genetic mitochondrial diseases that typically involve multiple organ systems and manifest with highly heterogeneous clinical symptoms [211, 212].

While MTx holds potential for enhancing cellular metabolism, its efficacy may vary by mutation type. In mtDNA‐related disorders, MTx could introduce functional mitochondrial genomes to dilute mutated mtDNA, possibly offering long‐term benefits beyond metabolic enhancements if the transferred mitochondria carry intact genomic material capable of replacing faulty mtDNA. Conversely, disorders arising from nDNA mutations present additional challenges. Although MTx may provide improvements to mitochondrial function, the transplanted mitochondria might be affected by the dysfunctional cellular environment created by the nuclear gene defect, potentially limiting the long‐term efficacy of MTx. Consequently, more frequent administrations or adjunctive therapies might be required to maintain mitochondrial functionality and achieve sustained therapeutic effects. Despite these challenges, the unique characteristics of MTx position it as a promising therapeutic approach that warrants systematic investigation in genetic mitochondrial disorders. The following sections review current research progress, organized by the genetic basis of disease, and discuss the unique challenges and future directions for clinical translation of MTx in this patient population.

### 4.1 Current Research Progress of MTx in Mitochondrial Genetic Disorders

Research on MTx for genetic mitochondrial diseases remains in its early stages, with a few studies conducted in preclinical in vitro and in vivo models to explore the feasibility, efficacy, and therapeutic mechanisms of this approach [213, 214, 215]. While preliminary results have been encouraging, challenges persist, particularly in developing appropriate animal models that accurately recapitulate the complex pathophysiology of human genetic mitochondrial diseases. Current research priorities include understanding the mechanisms underlying MTx therapeutic benefits, evaluating the extent of mitochondrial integration and persistence, and optimizing delivery methods for targeting specific affected tissues and organs.

#### 4.1.1 MTx for Nuclear DNA‐Based Disorders

**FIGURE 4:**
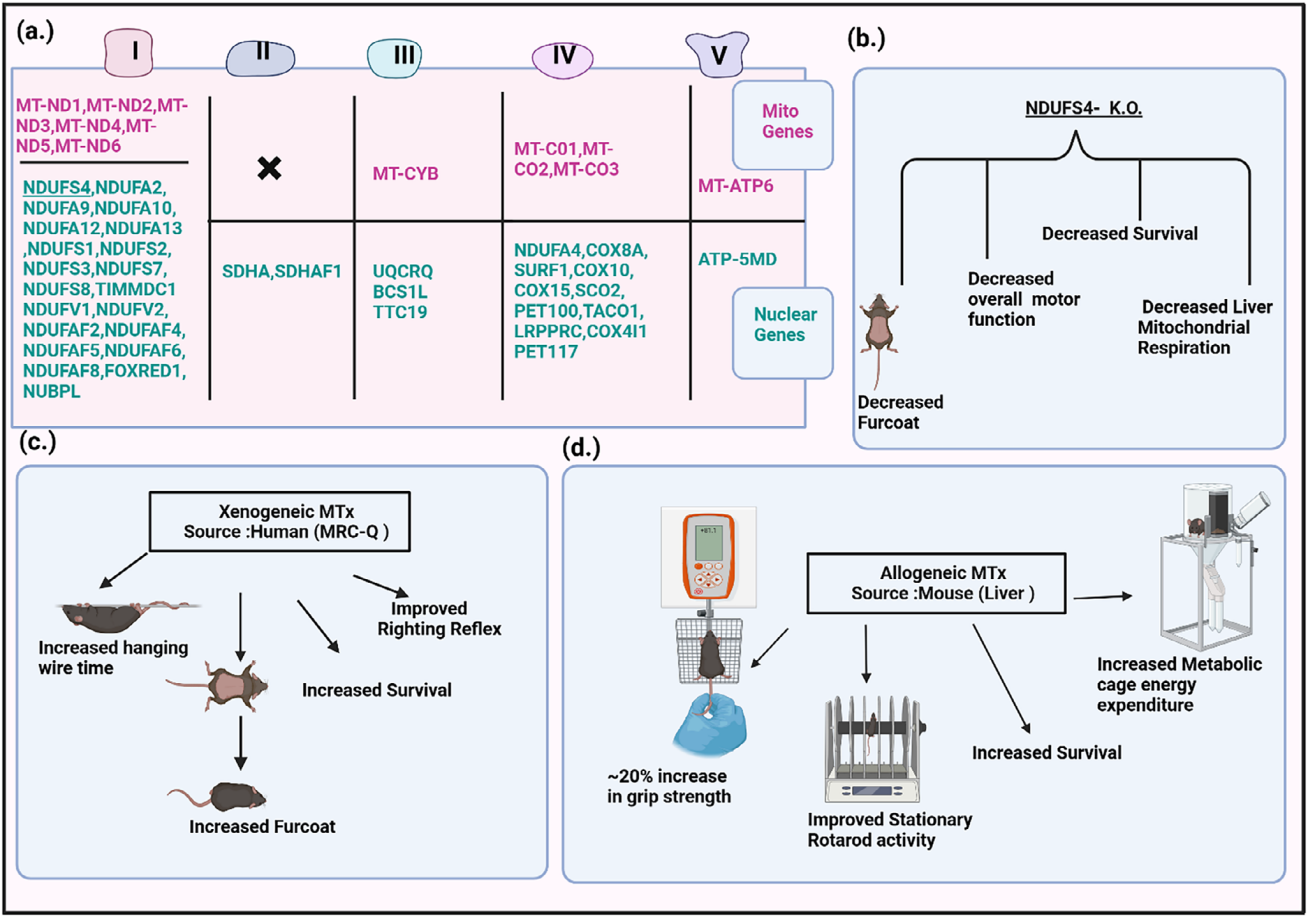
Genetic basis of Leigh syndrome and therapeutic effects of mitochondrial transplantation in NDUFS4 knockout mice. (a.) List of mitochondrial and nuclear DNA genes implicated in Leigh syndrome, categorized by their association with mitochondrial respiratory chain complexes I–V.(b.) Effects of *Ndufs4* knockout (KO) on mice, including phenotypic and metabolic alterations.(c.) Xenogeneic mitochondrial transplantation using HeLa cell–derived human mitochondria organelle complex‐Q (MRC‐Q; LUCA Science) and its observed effects.(d.) Effects of allogeneic mitochondrial transplantation with mitochondria derived from wild‐type mouse liver.

For disorders caused by nDNA mutations, MTx must function within a cellular environment where the host cell's nucleus continuously produces defective or missing versions of essential mitochondrial proteins, thereby rendering the nuclear‐encoded mitochondrial machinery dysfunctional and presenting unique therapeutic challenges. Despite these complexities, early preclinical work is beginning to demonstrate promising advancements. For instance, an in vitro investigation involving differentiated SH‐SY5Y neurons bearing the POLG p.A962T mutation found that the mutation was associated with increased mitochondrial ROS, dissipation of mitochondrial membrane potential, and depletion of mtDNA‐encoded subunits of respiratory chain complexes I and IV. Pep‐1–mediated MTx reversed these defects by restoring mitochondrial membrane potential, mitochondrial ATP levels, and mtDNA content, highlighting the neuroprotective potential of MTx in mitochondrial diseases [216]. These in vitro results are further complemented by a significant in vivo breakthrough presented by Nakai et al. in 2024, focusing on MTx and its therapeutic potential for Leigh syndrome using NDUFS4 knockout mice, a well‐established model with phenotypic similarities to the human condition (Figure 4). This study examined two therapeutic strategies: bone marrow transplantation and autologous and xenogenic MTx [213]. The study demonstrated that wild‐type marrow engraftment improved survival, neuromotor performance, and energy expenditure, with fluorescent tracking indicating systemic mitochondrial transfer into various cell types. Additionally, the intraperitoneal delivery of wild‐type liver mitochondria enhanced grip strength, rotarod coordination, survival, and energy metabolism, although body weight and thermoregulation remained unchanged. Xenogeneic transplantation using commercial HeLa‐derived human mitochondria (MRC‐Q, LUCA Science) achieved similar results. Importantly, therapeutic effects were observed only when healthy, functional mitochondria were used. Although the study did not fully examine the molecular mechanisms involved, it underscored the importance of using healthy, functional exogenous mitochondria, establishing MTx as a promising treatment avenue for Leigh syndrome. However, a critical aspect for the long‐term feasibility of MTx in nDNA disorders, highlighted by this study, is the duration of therapeutic effects and the optimal frequency and dosing needed for sustained benefits.

An in vitro MTx approach has also been explored to investigate its therapeutic potential in BTHS ‐derived cardiomyocytes. Barth patient‐derived cardiomyocytes display pronounced defects in mitochondrial function, including reduced oxidative phosphorylation capacity and impaired biogenesis. Transplantation of allogeneic mitochondria isolated from healthy donor iPSC‐derived cardiomyocytes not only restored mitochondrial respiratory function and ATP production but also improved mitochondrial network integrity and dynamics. Furthermore, MTx promoted structural and functional maturation of Barth cardiomyocytes, suggesting that targeted mitochondrial replacement can partially rescue disease‐associated cellular phenotypes and support cardiac development [217]. Given these promising in vitro results and the availability of multiple BTHS mouse models [218], further in vivo investigation is clearly warranted.

#### 4.1.2 MTx for Mitochondrial DNA‐Based Disorders

In contrast to nDNA disorders, mtDNA‐based diseases may be particularly amenable to MTx, as introduction of healthy mitochondria with wild‐type genomes could directly address the genetic defect through a potentially curative heteroplasmic shift. However, direct genetic modification of mtDNA in mammalian cells remains technically challenging, making it difficult to generate animal models that carry the specific pathogenic mtDNA mutations found in human disease. This represents a major obstacle in advancing MTx to in vivo studies for mtDNA disorders. While numerous mouse models have been developed for nDNA disorders, existing mtDNA murine models focus on general processes such as maintenance, transcription, and translation, rather than replicating specific pathogenic mtDNA mutations [219, 220, 221, 222, 223]. Consequently, these models often fail to recapitulate the complex pathophysiological conditions observed in human mtDNA diseases, which has largely limited research into MTx for mtDNA mutations to in vitro studies.

Despite this limitation, in vitro studies have demonstrated proof‐of‐concept for MTx in mtDNA disorders. Indeed, in rotenone induced cellular and mouse models of LHON, PARKIN mRNA–loaded nanoparticle–engineered mitochondria demonstrated therapeutic efficacy by restoring mitochondrial quality and complex I function. In vitro, mNP‐Mito was internalized predominantly via macropinocytosis, and delivery of exogenous healthy mitochondria increased the proportion of functional mitochondria with intact complex I, while concurrently promoting PARKIN‐mediated mitophagy to eliminate dysfunctional mitochondria. In vivo, intravitreal administration of mNP‐Mito significantly enhanced retinal complex I activity and restored ATP production. These improvements were accompanied by functional benefits, including a significant increase in head movements, indicative of improved visual performance. Importantly, no proinflammatory responses associated with allogeneic MTx were observed [47]. In a MELAS patient‐derived neuronal model, highly purified mesenchymal stem cells delivered exogenous mitochondria into neurons through endocytosis‐mediated transfer more efficiently than conventional mesenchymal stem cells. This mitochondrial delivery restored respiratory function, enhanced cellular bioenergetics, and sustained elevated ATP production for at least 21 days [214]. A separate study employed the cell‐penetrating peptide, Pep‐1, to facilitate mitochondrial transfer into cybrid cell models harboring the MELAS m.3243A>G mutation. Donor mitochondria were successfully internalized, leading to increased wild‐type mtDNA content, restoration of respiratory function, improved biogenesis, and reversal of abnormal mitochondrial morphology while protecting cells from oxidative stress [215]. Similarly, in cybrids harboring the MERRF m.8344G>A mutation, Pep‐1‐conjugated mitochondria were efficiently internalized and persisted for up to 15 days, restoring mitochondrial membrane potential, oxygen consumption, and ATP levels, while reducing oxidative stress. These effects were accompanied by upregulation of mitochondrial biogenesis markers and modulation of fusion‐fission dynamics, despite no significant change in mutant heteroplasmy [190]. This latter observation is critical, indicating that even without a complete heteroplasmy shift, metabolic improvements can occur.

While these in vitro findings are promising, their translation to clinical applications requires in vivo validation. This necessitates the development of animal models that accurately recapitulate human mtDNA mutations. Emerging technologies such as double‐stranded DNA deaminase A‐derived cytosine base editors (DdCBE) offer promising approaches for creating pathogenic mtDNA variants in vivo [224], potentially enabling generation of more accurate mtDNA mutation models that are essential for robustly evaluating the long‐term efficacy and safety of MTx.

### 4.2 Translational Challenges and Safety Considerations

While the preclinical evidence reviewed above demonstrates the therapeutic potential of MTx for genetic mitochondrial diseases, its translation to clinical application faces several unique and interconnected challenges that extend beyond the general technical considerations of mitochondrial isolation, purity, and storage. A primary concern is the inherent long‐term therapeutic challenge, as MTx can provide transient support but often cannot address the primary genetic cause of mitochondrial dysfunction. Consequently, achieving sustained therapeutic effects over extended periods is essential but complex. These challenges include genetic and immunological incompatibility, the necessity for prolonged mitochondrial persistence and effective host cell integration, and the ethical considerations exacerbated by repeated administration in the context of progressive pathology.

#### 4.2.1 Genetic and Immunological Incompatibility

A fundamental challenge arises from establishing initial genetic and immunological compatibility when introducing exogenous mitochondria. Autologous MTx is often not viable because the patient's own mitochondria harbor disease‐causing mutations. This necessitates the use of allogeneic sources, which introduces significant considerations. Mitochondrial‐nuclear mismatch is a primary concern [225]; reliance on allogeneic mitochondria introduces the risk of the transferred organelles not harmonizing optimally with the host cell's nuclear‐encoded proteins. Indeed, their optimal function requires harmonious interaction with the host cell's existing machinery. In disorders where the recipient's nuclear genome encodes defective mitochondrial components, the transplanted mitochondria might still be subject to the same dysfunction or operate suboptimally within that environment.

Immunogenicity and host response also remain a critical safety concern with allogeneic transplantation. In a uniquely comprehensive study, mitochondria isolated from 13 distinct species demonstrated viability across phylogenetic distances [226]. Remarkably, despite the xenogeneic approach, co‐culture experiments suggested minimal immune activation, with no significant elevation in inflammatory cytokine levels. Their findings implied that metabolically compatible mitochondria, even from phylogenetically distant sources, could be safely transplanted and yield enhanced therapeutic benefits due to preserved metabolic alignment [226]. However, immune responses in preclinical studies have been inconsistent: the McCully group observed no immune reaction in animals receiving autologous or allogeneic mitochondria, while the Brennan group reported that allogeneic mitochondria can activate human endothelial cells in vitro, elevating adhesion molecules, inflammatory cytokines, and chemokines [181, 227]. Moreover, circulating extracellular mitochondria in deceased organ donation have been associated with allograft dysfunction, implicating inflammatory signals as key determinants of MTx outcomes [181, 227, 228, 229]. This conflicting data underscores the need for a more comprehensive understanding of the immune response to exogenously delivered mitochondria, especially in diverse tissue contexts and with repeated administration.

Despite these complexities, clinical studies with MTx, though limited, have been encouraging from a safety perspective. As discussed previously, clinical studies to date have not reported major adverse events, supporting a preliminary favorable safety profile for MTx [21, 22, 186, 187]. However, these early trials are often small‐scale and short‐term, primarily assessing acute safety. The absence of serious adverse events in early clinical trials is encouraging but does not eliminate the need for careful monitoring of immune responses, particularly in the context of chronic, repeated administration. Systematic assessment of immune activation markers, mitochondrial‐specific antibody formation, and long‐term engraftment dynamics will be essential as MTx advances toward clinical trials in genetic mitochondrial diseases.

#### 4.2.2 Long‐Term Persistence and Host Cell Integration

Beyond the initial acceptance by the host, the long‐term therapeutic success of MTx hinges on the persistence and functional integration of transplanted mitochondria within recipient cells. Mitochondria are dynamic organelles with an active turnover rate. For sustained therapeutic benefit, transferred mitochondria must integrate effectively, establishing functional connections with the host cell's existing mitochondrial network or metabolic pathways. They must also survive and replicate, persisting over time, potentially through replication or by being continuously supplied, particularly in tissues with high metabolic demand and rapid cell turnover. Furthermore, they need to overcome host cell dysfunction. In nDNA‐based disorders, for instance, the survival and optimal function of transferred mitochondria may be compromised by the recipient cell's intrinsic inability to produce essential nuclear‐encoded mitochondrial proteins or by a toxic cellular environment due to increased oxidative stress or accumulating harmful metabolic byproducts. This directly impacts the potential for durable therapeutic responses, as transferred mitochondria may be degraded or become dysfunctional relatively quickly.

#### 4.2.3 Challenges of Repeated Administration and Progressive Pathology

The progressive and multi‐organ nature of most inherited mitochondrial disorders further complicates MTx therapy by requiring repeated systemic delivery to sustain therapeutic benefit. Repeated allogeneic MTx presents several interrelated challenges: First, immunogenicity becomes an escalating concern; with each administration, the risk of immune responses increases, potentially reducing efficacy and compromising patient safety [227]. This risk is amplified by donor‐recipient mismatch. Second, repeated invasive procedures increase patient burden and may limit treatment adherence, impacting overall therapeutic success. Third, targeting critical tissues such as the central nervous system or skeletal muscle becomes progressively more complex with each administration, particularly as disease pathology evolves. Fourth, determining optimal dosing frequency is challenging because therapeutic needs shift with disease progression and treatment response, requiring highly individualized and adaptive treatment plans.

#### 4.2.4 Ethical Considerations

As MTx progresses toward clinical translation, several ethical considerations warrant careful deliberation. These include the source of mitochondria (e.g., allogeneic human cells, xenogeneic sources, or engineered mitochondria), which raises questions regarding consent, safety, and potential long‐term biological consequences. Genetic identity and chimerism are also considerations; while MTx typically does not alter nDNA, the introduction of foreign mtDNA raises questions about genetic chimerism at the mitochondrial level and its long‐term implications. A thorough and transparent risk‐benefit assessment is paramount for a novel and invasive therapy, particularly for vulnerable patient populations like those with severe pediatric mitochondrial diseases, including discussion of potential for transient benefits versus long‐term cures and managing patient expectations. Lastly, access and equity will be crucial, as the cost and accessibility of such advanced therapies could raise significant ethical questions about equitable access to treatment.

## 5 Conclusions

The exploration of MTx signifies a transformative shift in the therapeutic landscape for genetic mitochondrial diseases. By directly addressing the underlying dysfunction of mitochondria rather than focusing solely on symptomatic relief or secondary manifestations, MTx offers a novel and potentially therapeutic approach. This strategy entails the introduction of healthy, functional mitochondria into affected cells, aiming to restore bioenergetic stability and mitigate oxidative stress. While research is still in its nascent stages, preclinical studies and emerging clinical evidence highlight the promising potential of MTx across a spectrum of mitochondrial dysfunctions. Continued advances in this field hold the promise of significantly enhancing patient outcomes, transforming lives affected by these debilitating diseases, and establishing MTx as a pioneering option in mitochondrial therapies.

## Conflicts of Interest

The authors declare no conflicts of interest.

## Data Availability

The authors have nothing to report.
